# Design and Implementation of a Mixed IoT LPWAN Network Architecture

**DOI:** 10.3390/s19030675

**Published:** 2019-02-07

**Authors:** Jesus Rubio-Aparicio, Fernando Cerdan-Cartagena, Juan Suardiaz-Muro, Javier Ybarra-Moreno

**Affiliations:** 1 Department of Information Technologies and Communications, Technical University of Cartagena, 30202 Cartagena, Spain; fernando.cerdan@upct.es; 2 Department of Electronics Technology, Technical University of Cartagena, 30202 Cartagena, Spain; juan.suardiaz@upct.es; 3 Hidrogea, Gestión Integral Del Agua S.A., 30008 Murcia, Spain; xybarra@hidrogea.es

**Keywords:** IoT architecture, IoT, LPWAN, Smart metering, Smart cities, LoRa, Sigfox

## Abstract

IoT is much more than a large number of objects or customer devices connected to the Internet. IoT offers organizations many more opportunities than they can imagine. According to this, sooner or later they will probably choose to build their own IoT network. In this article, we review the technologies of IoT LPWAN Sigfox and LoRa. It can be considered the most important at present due to its ability to make the smart city possible. We also propose the development, deployment and implementation of a mixed IoT architecture LoRa-Sigfox composed of components based on open hardware and software. The architecture is evaluated in a real environment focused on remote monitoring of water meter devices.

## 1. Introduction

The “Internet of Things” (IoT) is gaining importance and increasing participation in every field worldwide. This trend does not only apply to people in their daily lives, but also becomes a determining factor for companies. What some years ago was a potential technology of enabling new digital business initiatives and operational improvements, has today transitioned to the implementation phase.

IoT creates an ecosystem [1] that enables organizations to reinvent the way in which they relate to their customers, suppliers, end users and other stakeholders. Also in how their industrial processes and their working time, offering information that will allow companies explore new markets, products and services. This allows, among other applications, remote attention and monitoring to measure behaviours and much faster responses to emergency situations, prevention in the handling of equipment, or management of real time information coming from supply chains about offer, demand and shipment to customers; and, in general, automation and management of all assets throughout their life cycle [2]. 

The Internet of Things is the fundamental technological component on which the paradigm of connected objects is based [3]. Any intelligent object is able to send and receive information through the Internet, thereby increasing its functionality or adding value to the one it already has. Endpoints of the Internet of Things will grow at a 32.9% compound annual growth rate (CAGR) by 2020, reaching an installed base of 20.4 billion units. There has been a massive growth in the number of endpoints and major technology disruptions in sensor, device, gateway and digital twin technologies. This is coupled with the growth of edge computing technologies, where computing and analytics move closer to the endpoint to enable more real-time use case [4].

At present there are a wide variety of wireless technologies such as WiFi, Bluetooth, 2G/3G/4G, RFID, ZigBee and many others that allow us to deploy an IoT network. However, the suitable technology will be chosen according to the needs of the design, since each technology has its own characteristics and adapts to different applications. Network coverage, energy consumption of devices and transmission bandwidth, are some of the factors to be considered [5].

The connection of such a large number of objects envisages new challenges for the networks supporting them, and must have a high scalability to absorb the expected exponential growth as well as simplicity in the registration of new devices. In this context, the objects must have an optimal consumption so that they can be installed in different places without the need for external power supplies. On the other hand, it is interesting that the signal transmitted by the endpoint can reach long distances without attenuating excessively. Also the network traffic will be based on the sending of small packages of information every certain time so it will not be necessary a large bandwidth. Neither the transmission speed nor the latency of the network will be critical parameters. Most important, it will be just the data arrives, regardless of how fast they do it. Under these premises, the network technology that best adapts to the described requirements is LPWAN (Low Power Wide Area Network) [6].

The LPWAN name includes wide area and low power networks specific for IoT applications. They allow the deployment of a large number of connected objects at a low infrastructure costs. They offer long-range connectivity to cheap, battery-operated devices, which require the transmission of small amounts of data at regular intervals of time over a long lifespan [7].

In this work we rely on two of the most popular LPWAN communication systems for IoT networks, such as Sigfox and LoRa.

LoRa from Semtech is similar in performance to Sigfox, but with a spread spectrum (SS) approach. Semtech focuses on chip manufacturing for both sensors and base stations, and its business model is based on allowing companies or individual users to deploy their own networks. In the work presented here, this feature is very interesting because it favours the use of M2M and relay communications in low coverage areas where devices have connectivity problems. The business model is different from Sigfox. The mobile telephony operators promoting LoRa technology, offer IoT as another company service.

Sigfox is a French startup that gives its name to technology. It is a solution that seeks compatibility with many module manufacturers adopting a neutral position such as Avnet, Telit, Texas Instrument, etc., and therefore there are more variety of devices compatible with this technology in the market known as Sigfox ready devices. Sigfox acts directly on the market as an operator and can talk directly with end customers, which is an opportunity for integrators and specialists in solutions and services to adopt this technology.

In addition to connecting a large number of objects, the variety of them is also very wide, with many different IoT categories including low-power devices, smart cars, wearables or smart meters. The latter collects user consumed water, gas, or electricity. Other sensors and devices send data from a variety of sources, such as temperature, light, sound, etc. The result of being such a broad term is that we will not find a universal architecture that meets the requirements and needs of each IoT category. An example of reference architecture for Smart meters can be found in [8] which covers multiple aspects such as a cloud architecture that allows monitoring, managing, interacting and processing data from IoT devices. Nevertheless a global reference architecture must also include a network model to communicate devices and have agents and codes for them, as well as the requirements of the products that are capable of supporting this reference architecture [9].

The management of the integral cycle of urban water, from the moment water is collected and arrives at the tap, until it is returned to nature and reused, is divided into three phases: supply, sanitation and reuse. The supply ranges from the collection of water until it reaches the connections and meters of the buildings. The sanitation is in charge of the used water, which leaves the houses, and it is returned respecting the environment. The reuse, which is carried out in some cases, uses water coming from for different users different from human consumption, such as garden irrigation, agriculture or some industrial uses. Water is necessary daily. Although it seems abundant, it is not an unlimited resource, and in particular drinking water is necessary for human life. Without conservation efforts, the water supply may be exhausted. Throughout the cycle there are agents in charge of pricing, to ensure customer service, data statistics and prepare regulations [10].

In this work, we propose the design, implementation and evaluation of a solution specialized in residential water management through the development and connection of smart water meter devices, integrated into a mixed IoT LoRa-Sigfox architecture. This solution will allow us to efficiently manage the water distribution network. Unlike most current remote reading systems, and making use of bi-directional communication, in addition to remote reading, will be possible to act remotely on the customer’s equipment interrupting the supply in case of incidents. Smart remote reading thanks to the use of smart meters facilitates greater control over the water consumed and thus savings in water consumption are achieved contributing to the development of the EU water directive [11] and the development of Smart cities in this sector [12].

During the implementation of the proposed solution, it was chosen to develop our own devices with embedded hardware and software. The reason was that commercial products did not have all the required functionality and additionally the following advantages have been achieved: (1) Customization capacity according to the needs of the proposal; (2) Scalability to add new functionalities and (3) Low cost design. Likewise, since it is an open design of both hardware and software, it facilitates the research community to create their own solutions, overcoming the drawbacks of other closed commercial products.

The smart IoT meter device developed, unlike similar products, not only performs the remote reading function, it is also capable of carrying out remote control over adjacent devices such as a solenoid valve. It was made using the minimum number of possible components and reduced cost, matching the needs of simplicity and economy. The use of a high-level language interpreted for programming facilitates customization. Regarding the connectivity to the network, given that in certain locations, especially inside buildings or basements, we may have absence or instability of link to the Sigfox network, a new device was built to be located in an intermediate position with good connectivity to Sigfox. This device sends to the network the data received from multiple smart meter units with bad or without connectivity. This intermediate position implements a point-to-point radio link based on LoRa technology which benefits from a wide range and high interference immunity. In this way we avoid the use of Sigfox micro access stations, saving important infrastructure costs. Finally, for the areas with LoRaWAN coverage, we deployed and implemented our own developed base station based on open hardware and software which acts as a gateway to IP.

The rest of the article is structured as follows: Section 2 reviews the LPWAN Sigfox and LoRa technologies. Section 3 focuses on a complete description of the proposed mixed network architecture. Section 4 is dedicated to the hardware elements developed: smart water meters with IoT connectivity, radio link for shadow zones and LoRaWAN base stations. Section 5 describes a case study and its performance evaluation. Finally, Section 6 includes the conclusions.

## 2. Related Technologies

### 2.1. LPWAN

As already mentioned in the introduction section, Low Power Wide Area Networks are presented as an optimal technology for IoT in cases where great autonomy, long range, low cost and transfer of small data amounts [13]. Unlike WiFi, Bluetooth, ZigBee or NFC, it allows longer range links and scalability is greater since supports a higher number of devices. In comparison with cellular technologies such as 2G, 3G or 4G, the energy consumption is lower, which increases the autonomy, the cost of the device is smaller and the connectivity of new devices is simpler [14].

NB-IoT is a LPWAN technology standardized by the 3GPP (3rd generation partnership project) which uses licensed frequency bands assigned to mobile telephony operators and takes advantage of its already deployed infrastructure. Although it is based on a simplified version of the LTE protocol, still requires more energy than other LPWAN solutions because the synchronous communication, QoS handling and orthogonal frequency division multiple access (OFDMA). Despite it has the highest throughput and lowest latency, additional drawbacks are the lowest range and not all suburban areas benefit from LTE coverage [15].

The LPWAN technologies which are currently receiving the most attention from the industry and the research community are Sigfox and LoRa. Both operate in ISM radio bands (Industrial Scientific Medical). In the ISM bands it is possible to operate without a license provided that the restrictions of radiated power, bandwidth and transmission cycle are met. The band most used in Europe by these technologies is the 868 MHz, defined in the ECC 70-03 [16]. Regulation for this spectrum segment allows an ERP (Effective Radiated Power) of 14 dBm at 27 dBm and an operating cycle below of 1% or 10%. This implies that the same device is not authorized to emit beyond 1% every hour on each frequency, that is to say 36 s, which depending on the duration of the transmission of the message, can suppose a maximum of 6 messages per hour, that is, a message every 10 min.

### 2.2. Sigfox

Sigfox [17] is not only the name of an IoT technology but also a network operator that provides a complete solution, which covers since the collection of data from objects in any area of the world under coverage up to the transfer of these data towards the information system of any possible customer. The business model is based on invoicing for the connectivity provided to the devices. Since 2009, Sigfox has had a rapid growth, and in November 2018 is available in 53 countries, with an estimated coverage area of 5 million square kilometres [18]. Currently, each base station reaches an area from 3 to 10 km in urban environments and from 30 to 50 km in rural areas, offering different services for up to 1 million devices [19].

Before a Sigfox device can be registered in the network, it must pass a certification process defined by Sigfox’s official specifications, thus serving to ensure compatibility and quality of service. In these certification tests, both the link layer protocol and the RF radiation performance are evaluated, giving rise to a classification based on the transmission power [20]. When the certification process is passed, an official certificate is provided, which will later be necessary for the registration of any other device of the same model. In addition, each device is associated with a pair of ID/PAC identifiers that are necessary for the registration in the Sigfox network. The ‘Device-ID’ is a globally unique 32-bit identifier that is recorded in the non-volatile memory of the device and it does not change during the lifetime of the device. The PAC (Porting Authorization Code) proves the ownership of the device and changes each time the device is registered or transferred.

**Figure 1:**
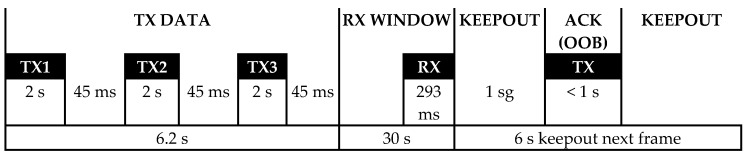
Chronology of a Sigfox communication.

For transmission of information, in the physical layer of the protocol is used a UNB (Ultra Narrow Band) radio technology, with a channel width of 100 Hz, DBPSK (Differential Binary Phase Shift Keying) modulation and a bit rate of 100 bps [21]. The modulation is the simplest one of the phase shift type, offering the highest immunity to noise due to the fact that the maximum difference of symbols is 180°. By concentrating the radiation in a very small bandwidth, the energy density is increased and a link more immune to signal interference with higher bandwidth and power is achieved [22]. It is possible to achieve long-range wireless links of up to 163.3 dB of total loss with 14 dBm of transmit power, using an antenna with 2.15 dBm gain, a base station receiver with a sensitivity of −142 dBm and a reception antenna of 5.15 dBm [23]. In Figure 1, it can be seen that each transmission period of the device lasts around 2 s and is repeated 3 times at different frequencies in order to increase the possibilities of reception by the network. The base stations maintain reception in a 192 KHz segment around the central frequency (868,130 MHz in Europe) and the devices can transmit on any frequency within that segment [24]. If a response is requested, a reception window of 25 s is opened and a confirmation of reception is subsequently generated. For the transmission from the network to the device, GFSK (Gaussian Frequency Shift) modulation is used at a speed of 600 bps and a channel is chosen according to the one used for uploading within the 200 kHz downlink segment and the European central frequency of 869,525 MHz [25]. Therefore, it is a bidirectional but asymmetric communication, which is always initiated by the device and only is produced a downloading communication if it is requested by this device. The number of uploading messages per device to the network is limited to 140 per day and the number of downloading messages is limited to 4.

**Figure 2:**
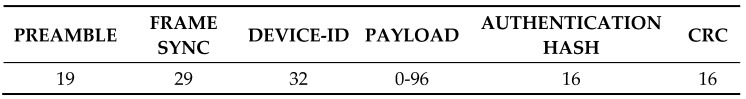
Uplink data-link frame. Fields and associated length.

In the data link level (MAC, Medium Access Control) associated with the OSI model (Open System Interconnection), each uplink frame has a total length of 26 bytes (208 bits). Up to 12 bytes (96 bits) of this data link frame can be a payload, that is, a message defined by the user. The protocol overload consists of a preamble for synchronization of the receiver, a device identifier for authenticating the message, a message identifier for detecting duplicated elements and a sum of bits for error checksum. It does not include any signalling with the network and messages are not confirmed either. The user is free to distribute as desired the information within the 12 bytes of payload, since these will be stored and will arrive at the client’s servers as is [26]. Figure 2 shows this frame corresponding to OSI level 2.

**Figure 3:**
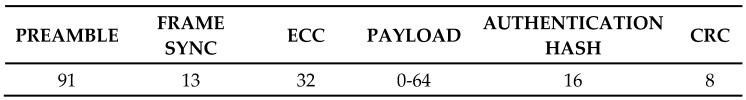
Downlink data-link frame. Fields and associated length.

The downlink frame in Figure 3 can contain up to 8 bytes of customizable information depending on the application. The content can be updated from the Sigfox backend with constants or variables such as the current date and time, or be generated dynamically from the user’s infrastructure.

Regarding security, the emitter is authenticated by means of the device identifier and the message authentication is performed by means of its sequence number. As a negotiation with the network, it is not necessary at the moment of sending messages, the device transmits the message without waiting for a response; therefore, trying to interfere with the receiver of the device does not avoid communication. In the radio link there is no encryption of the messages, this task is assigned to the OSI application layer if necessary. A VPN tunnel is established between the base stations and the Sigfox servers and the secure HTTPS protocol can be used to communicate with the user’s infrastructure.

The topology of Sigfox network is a ‘star of stars’ type, connecting multiple objects to the same base station and all the base stations to the Sigfox servers. The base station works as a sink of data received towards certain IP address. The detection of duplicated elements and the authentication are managed by the network server, not by the base stations. The concept of collaborative network is used. Each zone is usually covered by 3 base stations in what is called ‘spatial diversity’. With 3 base stations in 3 different locations covering each object increases the reliability of message reception.

### 2.3. LoRa

**Figure 4:**
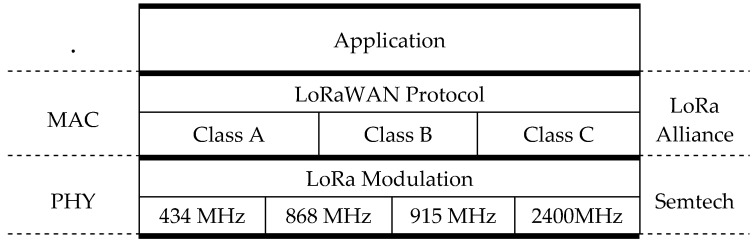
Layer model for LoRa and LoRaWAN.

LoRa (Long Range modulation technique) [27] is a solution for LPWAN networks based on spread spectrum transmission techniques and CSS modulation (Chirp Spread Spectrum). It was designed in 2010 by the French startup Cycleo, which was acquired by the semiconductor manufacturer Semtech Corporation in 2012, who is currently the owner of the technology, has registered the LoRa brand and maintains all rights. The business model consists of obtaining benefits for the LoRa radio chipset, leaving the final user or an intermediary with the deployment and management of the network for connected objects. The LoRa specification only describes the physical layer (PHY). As link layer (MAC) is available LoRaWAN (LoRa for Wide Area Networks). It is the open source proposal of the LoRa Alliance that can be used freely in own developments for both devices and gateways. Figure 4 shows the layered organization of LoRa and LoRaWAN. The LoRaWAN protocol includes encryption, authentication, adaptive speed and error correction [28].

The radio chip covers the frequencies between 150 and 960 MHz allowing operation in most ISM bands around the world. The LoRa modulation uses a signal called chirp that changes frequency continuously sweeping the entire width of the radio channel which is typically 125 KHz. By adjusting the Spreading Factor (SF), Bandwidth (BW) and Coding Rate (CR) parameters, a compromise solution can be reached between data rate, link budget, interference immunity and spectrum occupancy. Each bit of information is represented by multiple sweeps or symbols, and the relationship between the speed of symbol transmission and the duration of the sweep is the Spreading Factor, with typical values of 7 to 12 corresponding to bit rates of 11000 bps to 250 bps also called Data Rate (DR0-DR6). At higher SF values the effective transfer rate is reduced but the robustness of the link is increased. Also in order to increase it, cyclic error coding is used with possible values from 4/5 to 4/8 for detection and correction of errors assuming an overload ratio between 1.25 and 2. The use of a greater bandwidth allows increase speed, but also reduces sensitivity and is conditioned by the regulations of each region [29].

LoRa technology is able to demodulate signals 19.5 dB below the noise level, unlike most systems using FSK (Frequency Shift Keying) modulation that need a signal 8–10 dB above the noise level, being able to achieve distances of 6 km in urban environments and 18 km in rural scenarios with total link loss of up to 154 dB and receiver sensitivity of −142 dBm [30]. An added feature of LoRa is the ability to demodulate several signals simultaneously on the same frequency if they have different SF, thus creating different virtual channels and increasing the capacity of the gateway. In Europe, 3 mandatory channels are established for all the gateways in 868.100 MHz, 868.300 MHz and 868.500 MHz. In addition, others can be defined according to the operation policy of each network.

Three categories of devices are defined [31]:

- Class A: They are kept in energy savings most of the time. After sending information they maintain two reception windows and return to standby. They cannot receive data at any time.
- Class B: Communication at regular intervals by synchronization with a beacon. Although they do not need to send data, they wake up periodically to receive from the network.
- Class C: Continuous link to the network. They can receive and send data at any time.

**Figure 5:**
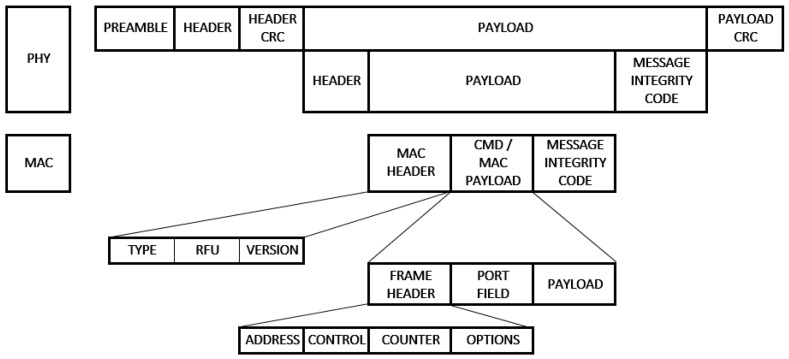
Uplink LoRaWAN frame and fields.

**Figure 6:**
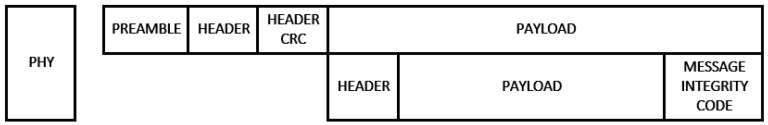
Downlink LoRaWAN frame and fields.

Figure 5 shows the fields that make up a LoRaWAN uplink frame both physically and at the MAC layer level. It can be seen in Figure 6 that the downlink frame does not include integrity checking at the physical level to keep messages as short as possible and reduce their impact on the limitations of the operation cycle in the ISM bands. The payload can be a maximum of between 51 and 222 bytes, depending on the SF and both in the uplink and downlink [32]. Each device has a unique and permanent 32-bit identifier called DevEui. LoRaWAN includes security and authentication based on the AES128 encryption scheme (Advanced Encryption Standard) and other security standards described in IEEE 802.15.4/2006. Unlike other systems that depend on a single key for both functions, LoRaWAN separates between authentication and encryption. The AppSKey key is used to encrypt the message payload. Authentication and message integrity control uses the network session key NwkSKey. There are two device association methods to the network [33]:

- ABP (Activation By Personalization): With this method it is necessary to program the security keys in the device. Based on the DevEui, a DevAddr, NwkSKey and AppSKey are generated for each client to be used during communication.
- OTAA (Over The Air Activation): In this case, the device already contains the information necessary to join the network and it is only necessary to register its DevEui and AppKey in the server. In each association request the device sends the DevEui and AppEui identifiers. The server generates the AppSKey, NwkSKey keys and sends them to the client next to the DevAddr to be used during the rest of the communication.

The network topology is a star of stars. Multiple devices can access through the same gateway and all the gateways are linked via IP to the network server. The gateway performs bridge functions by adding extra information such as the level of the received signal, but no association is required with it. Several gateways can receive and forward the information of the same object and it is the network server responsible for filtering duplicates, authenticating the devices and selecting the most suitable gateway for the downlink [34].

## 3. IoT Mixed Architecture

**Figure 7:**
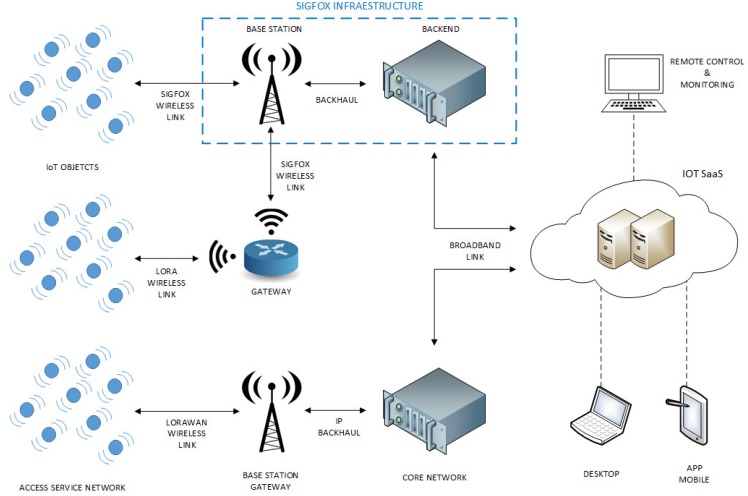
Proposed IoT mixed architecture.

The network architecture proposed and shown in Figure 7 combines a multiple access level for the information provided by the IoT endpoints. It is feasible to collect data both through the Sigfox network with its own established coverage, and through LoRaWAN networks already established or deployed for this purpose, as well as endpoints placed in locations without coverage of any of the previous networks. In this case, the link is made via intermediary devices that facilitate connectivity using LoRa technology.

The base stations deployed along a coverage area allow the exchange of information with the endpoints via a specific radio link for IoT. They do not perform any data processing, they just perform the gateway function to an IP network that links them to the network server through the backhaul. Two types are distinguished, those of the Sigfox network, managed by the same operator and our developed base stations to provide LoRaWAN coverage.

The network server or Core Network performs the functions of device management (registration, authentication and management of data traffic). Depending on the quality of the link, it can ask the devices for readjustments in the speed transmission and bandwidth. Regarding the received frames, it verifies its integrity and discards duplicates. It is also responsible for the management of gateways, maintaining a database with those registered in the network and selecting the most appropriate to transmit the download information according the received signal level. In this mixed architecture, this means that two network servers coexist. The first of them manages the traffic of the LoRaWAN base stations. The second, integrated in Sigfox, manages Sigfox base stations and provides the received payload from endpoints. Finally, all data are forwarded to the IoT SaaS through the broadband link.

The IoT SaaS (Software as a Service) carries out the management of the database that contains the messages received from all the connected objects. It uses big data techniques and it can also be equipped with business intelligence techniques for the processing of such data. In addition, it contains the necessary backend programming so that users can access information by both desktop and mobile devices in a friendly way.

## 4. Electronic Design

### 4.1. Description

Some hardware devices have been developed and built in order to meet the needs experienced in the deployment of the network and that were not covered by other commercially available products. Also working with open-software and open-hardware provides freedom to implement the required functionality and expand it in the future. Hardware developments are a residential water meter data acquisition device, a link device for shadow areas and a LoRaWAN base station.

### 4.2. Meter Data Acquisition and IoT Connectivity

The device described here is intended to collect data from residential water meters and send them to an information infrastructure over a specific IoT radio link.

The water consumption data is acquired in the form of electrical impulses whose frequency depends of the current flow and is generated either by the meter itself if it is remote metering ready, or adding to the meter a module which detects the needle movement and generates the impulses.

Unlike other commercially available solutions, this unit is also capable of performing remote control on an external electrical device through a potential-free output. An application example could be acting on a solenoid valve and in this way take control over the subscriber’s water supply service. To receive the remote control command the IoT downlink is used and once the new condition is established, no additional energy supply is required to hold that state.

**Figure 8:**
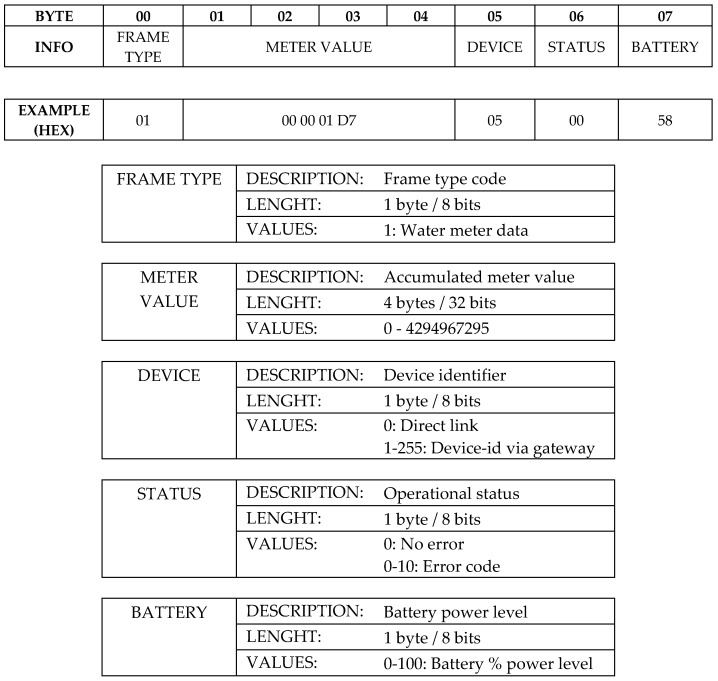
Uplink frame and fields description.

IoT connectivity capabilities are multiple and links can be established to the Sigfox network, any available LoRaWAN network, or to the own gateway device described in point 4.3 using LoRa modulation and enabling connectivity in shadowed areas without direct link to the aforementioned networks. The data frame sent to the IoT network has a specific format defined for this application and shown in Figure 8. The first field named Frame Type conditions the rest of the frame both in length and in all other defined fields. In this way, and although a particular frame type for this application is described below, it is easily adaptable to other cases and ready to future improvements only setting a new Frame Type. The maximum frame length will depend on the used network, being currently Sigfox the most restrictive with a maximum uplink payload of 12 bytes. The next field is the accumulated consumption value. Due a typical residential water meter shows the accumulated value in 8 number wheels and therefore a range from 0 to 99999999 litres, the field size has been set to 4 bytes (32 bits) which means it is able to represent unsigned values from 0 to 4.294.967.296, enough for this case. Following, a device identification byte is present to distinguish between 256 units using the same gateway described below in Section 4.3. The status field is a byte containing an error code for remote fault diagnosis. At the end, the battery field carries the power level of the battery. In the example shown in Figure 8, the device number 5 sends a water consumption value of 471 litres without error and with a remaining battery power of 88%.

**Figure 9:**
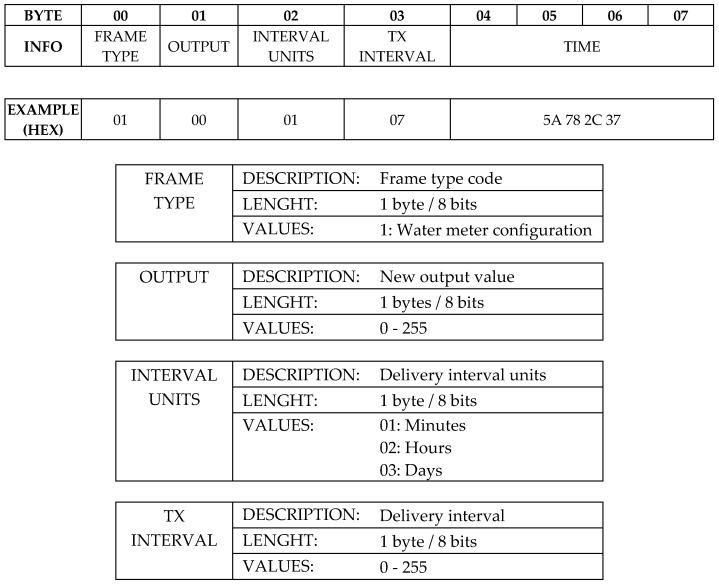

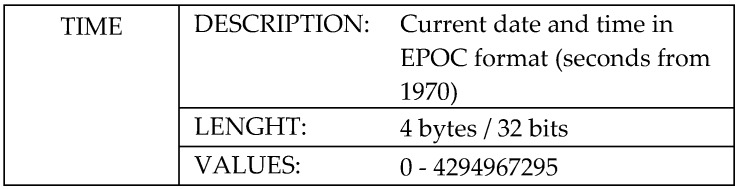
Downlink frame and fields description.

Optionally, and using the downlink from network to device, a frame with the format shown in Figure 9 can be received. It contains the new output value for remote control, data delivery interval and current date and time to update the internal RTC (Real Time Clock). The first frame field named Frame Type determines the other fields in the frame. For this particular application, the frame shown in Figure 9 has been set up, but it can be adapted to other cases setting a new Frame Type. The maximum frame length will depend on the used network, being currently Sigfox the most restrictive with a maximum downlink payload of 8 bytes. In this application, the device output is digital and can be set to two possible states: activated or deactivated, but the Output field has been defined as a byte (values from 0 to 255) in order to enable controlling analog loads using PWM (Pulse Width Modulation). For instance, a solenoid valve capable to adjust its aperture to deliver the fluid at different flow rates. In the example shown in Figure 9, the output is deactivated, the delivery interval is set to 7 min and the current time is supplied.

**Figure 10:**
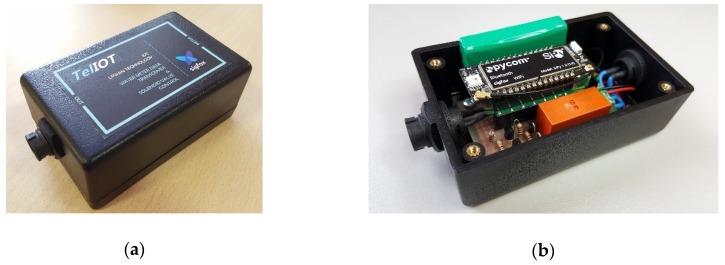
Data acquisition device with IoT connectivity: (**a**) External view; (**b**) Internal view.

The device showed in Figure 10 has been developed, and includes an electronics circuit board where are embedded the microcontroller and communications module, discrete electronic components to acquire the impulse signal generated by the water-meter and the voltage level of battery, jointly with a bi-stable relay to control external loads. The assembly is available in a plastic housing of ABS material with IP54 protection and threaded industrial connectors for the meter and the solenoid valve. No external antenna or charging port are necessary.

Electronic design is based on the Pycom LoPi4 [35] module for IoT solutions. The Pycom [36] module includes the Espressif ESP32 chipset as main processor running a MicroPython [37] interpreter. This offers a combination of power, friendliness and flexibility. It also includes multiple ways of connectivity: WiFi, Bluetooth LE, Sigfox, LoRa/LoRaWAN and FSK. WiFi and Bluetooth 2.4 GHz antenna is integrated in the module but it requires an additional one for the 868 MHz band in order to use Sigfox/LoRa capabilities. Due to this, it has been glued inside the box an 868 MHz flexible antenna with small dimensions and UFL connector suitable for the LoPi4 module.

High frequency noise filtering is applied to the signal coming from the water-meter. An I/O port of the microcontroller is used to acquire it, which has been configured as digital input and has been activated the capability of generate an interruption to wake up the processor from the low power consumption mode.

In this prototype, power is supplied by an included rechargeable Ion-Lithium battery, but due to the low consumption characteristics of the circuit, it is not needed to recharge it in several years, so it could be used a primary non-rechargeable Lithium Thionyl Chloride battery as an improvement making the cost of the product even cheaper. Battery voltage range (3.4–4.2 V) is directly compatible with the input power range in the Pycom LoPy4 (3.4–5.5 V), avoiding the need for additional components. 

To increase load control flexibility, a potential free output has been used, operated by a relay able to manage 220 VAC loads. To accomplish the large autonomy requirement desirable in any IoT device operated on battery, a dual coil bi-stable relay has been adopted. This relay is energized during 30 ms to open or close contacts and is able to remains in the last condition without continuous energy consumption. As relay coils require higher current to the one supplied by the microcontroller, outputs current drivers have been added. Although the chosen relay is a 3-way type, in this application relay contacts are used as a simple switch to open or close an external electrical circuit connected to the solenoid valve.

**Figure 11:**
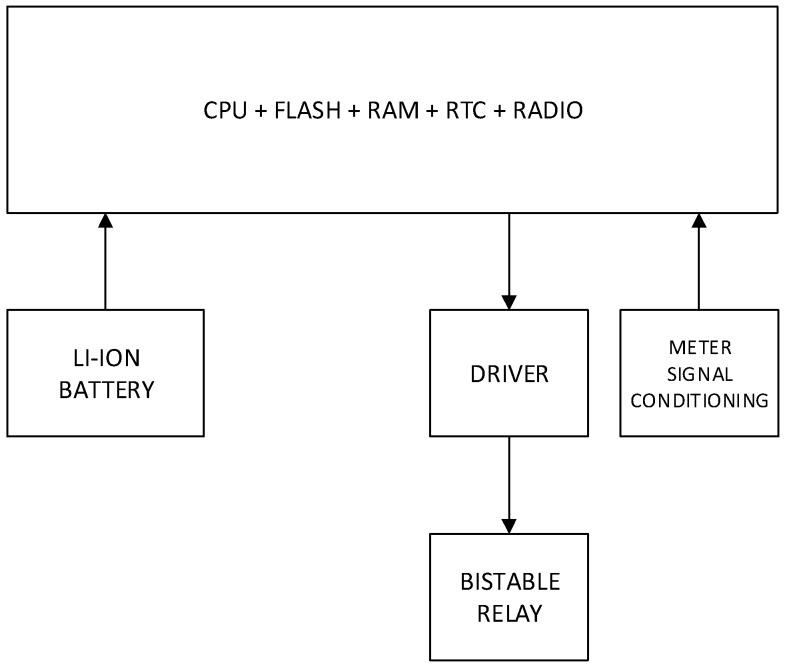
Block diagram of the data acquisition device.

In Figure 11 is showed a block diagram with all the elements of the design described here.

**Figure 12:**
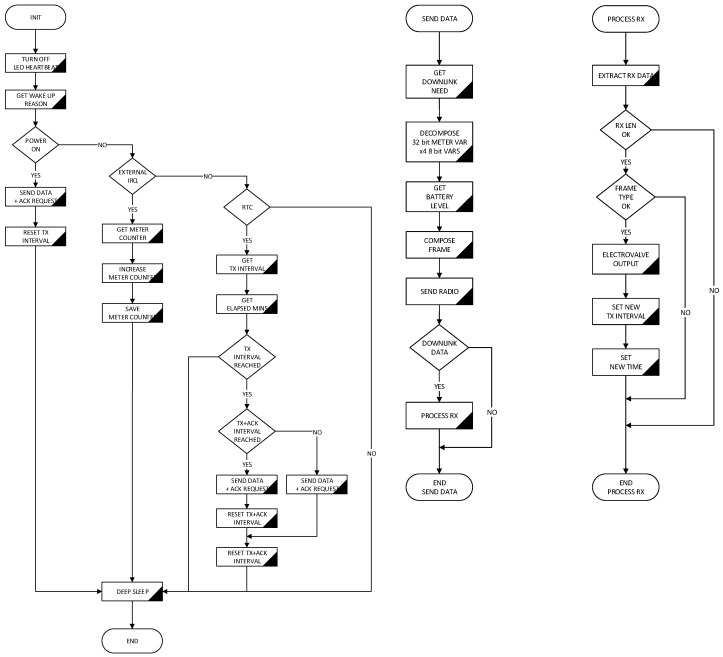
Flowchart of embedded software in water-meter device.

Device operation, as shown in the flowchart in Figure 12, starts in a low power state until meter activity is detected. Then water consumption value stored in non-volatile memory is updated and low power mode is set again. If user defined data delivery interval is reached, the frame with the information to be sent is generated, radio hardware is initialized and it proceeds to the transmission. In the Sigfox network case, the maximum allowed uplink and downlink messages per day are taken into account, so a downlink to receive new device configuration is only requested once a day. If output status change or other configuration data are received, they are executed and power mode is set again. Both the way of operation and the selection of hardware components allow an energy autonomy of 2 years minimum with the internal battery. Autonomy variation will depend fundamentally on the configured delivery interval, since it is the transmission process the one that consumes most of the energy.

**Figure 13:**
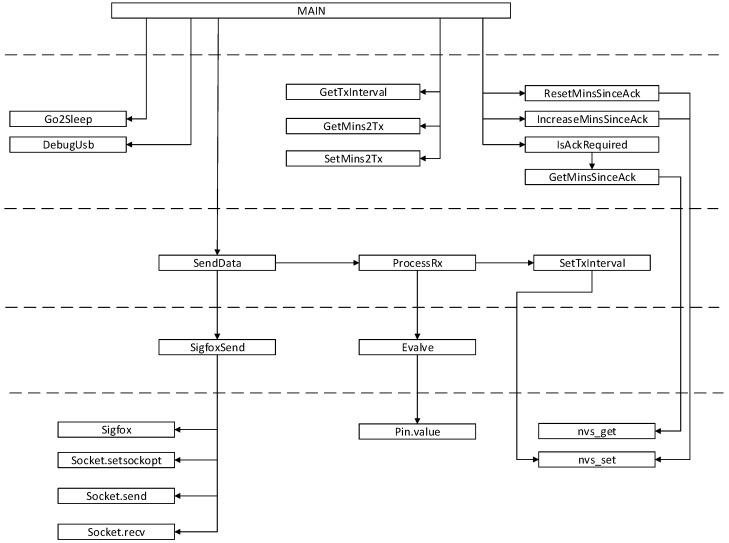
Embedded software functions diagram by level.

Embedded software has been structured in layers so it is scalable and easily adaptable to different types of input signal and multiple IoT radio technologies. As can be seen in Figure 13, all the specific code to the Sigfox radio in this case, is encapsulated in the lower layer, so only replacing this one, the software can be easily adapted. For the same hardware, several versions of the embedded software have been developed enabling connectivity to Sigfox, LoRaWAN and LoRa according to the customer’s needs. There is plenty of room in the microcontroller program memory to implement a multi-protocol version without hardware changes.

### 4.3. Gateway Intended to Areas without Sigfox Network Coverage

**Figure 14:**
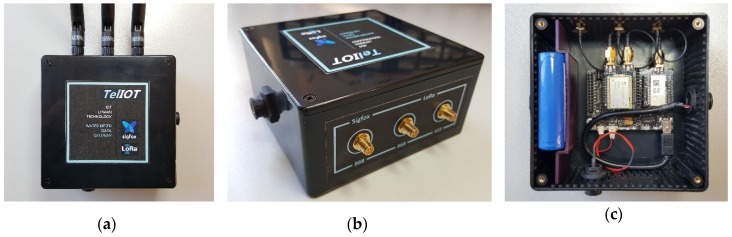
Gateway device to enable link in locations without Sigfox network coverage: (**a**) External view; (**b**) Antenna connectors; (**c**) Internal view.

The device described here and shown in Figure 14, allows solving link faults detected in some locations without Sigfox network coverage. Installed in an intermediate location with suitable Sigfox network coverage and with a point to point LoRa link to the meter devices, it is able to send to the network the data from multiple meters. In addition to enabling the link, there is a saving in the number of network access licenses, since only the bridge device needs one. In the link to the meters, LoRa is used in physical layer due it is an IoT radio technology that allows long range and immunity to interference without sacrificing consumption. In addition, the link is possible in both 868 MHz and 434 MHz ISM bands.

**Figure 15:**
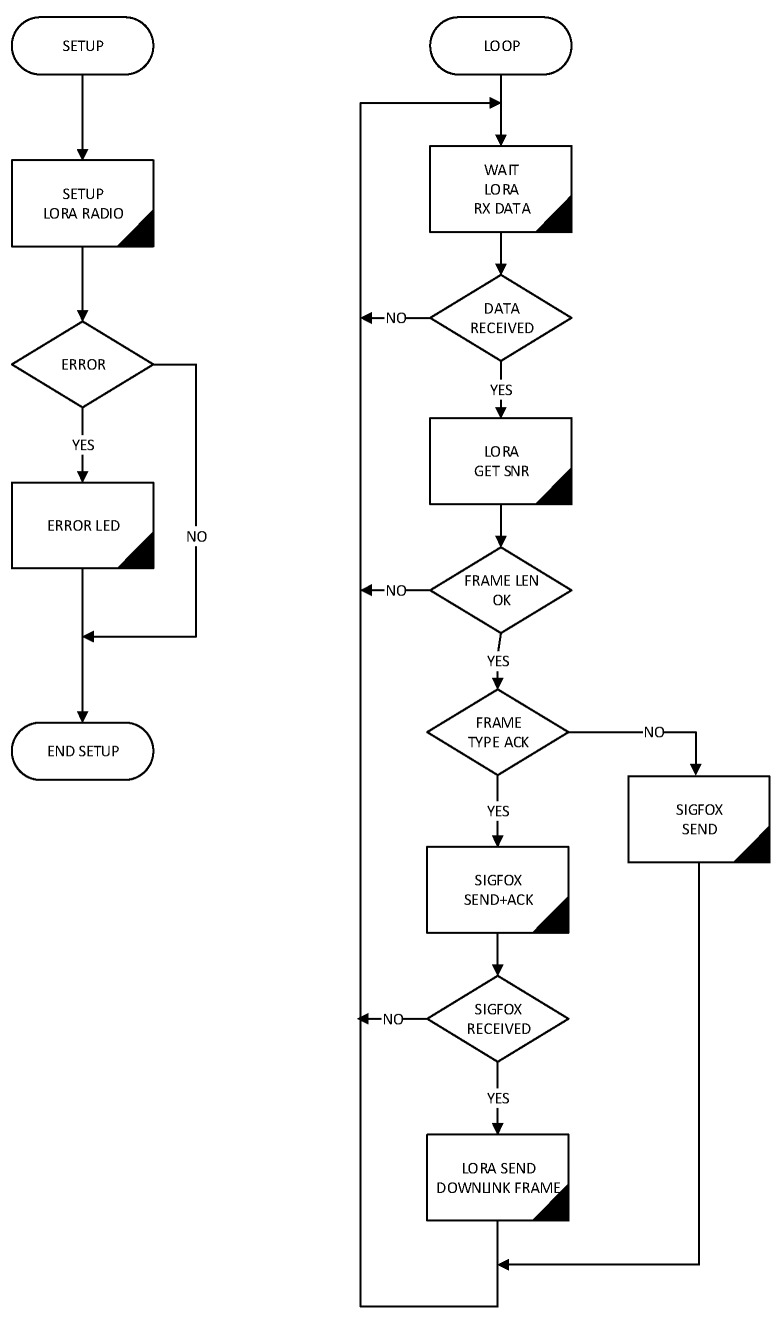
Flowchart of gateway embedded software.

As can be seen in the flowchart of Figure 15, device operation has a waiting state until a water-meter data frame is received by the LoRa radio module. If a frame is received and its format is validated, it is stored in a pool in non-volatile memory to be sent later to the Sigfox network at the delivery interval established.

**Figure 16:**
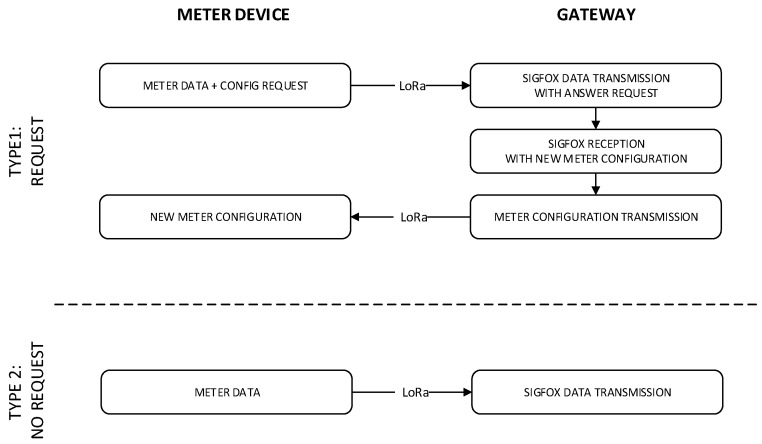
Operation diagram using the gateway.

This gateway device is compatible with the meter device frame format already described in the previous Section 4.2. Figure 16 shows the full operation diagram of both the counter and the gateway. There are two possible cases. In the first one, corresponding to a type 1 frame, the consumption data is sent from meter to the gateway using the point to point LoRa radio link and new configuration parameters are requested. Upon receiving a frame of this type, the gateway forwards the data to the Sigfox network requesting a downlink answer. This message will contain the configuration parameters that the user has previously established and it will be forwarded to the meter device via LoRa. The meter device will update operation parameters described in Section 4.2. In the second case, corresponding to a type 2 frame, the counter device only sends the consumption data without waiting for a response. The gateway forwards this data to the Sigfox network without requesting a downlink message.

The device consists of a Waspmote motherboard [38] where are located the microcontroller, the non-volatile memory, a real-time clock (RTC), voltage regulator and battery charge controller. These main board servers are a physical support and electrical interconnection for two communication modules, the first of them complies with LoRa radio specification and the second one with Sigfox radio and link layer protocol specification. Both radio modules include antenna connector, so using coaxial cable this signal has been feed to SMA type connectors placed outside the box enabling three external antennas to increase the link range, one of them for Sigfox in 868 MHz band and two for LoRa in 868 and 434 MHz bands. USB port of the main board is also available outside the box using an industrial type connector. In this way, the device can be powered, recharge the internal battery or perform firmware updates. The built-in 6600 mAh Ion-Lithium battery allows maintaining the operation in case of lack of external power supply. It is also possible to perform autonomous operation without external power supply connecting a solar panel with voltage range of 6–12 V and minimum current of 280 mA. Built-in battery will be charged using the power supplied by the solar panel.

**Figure 17:**
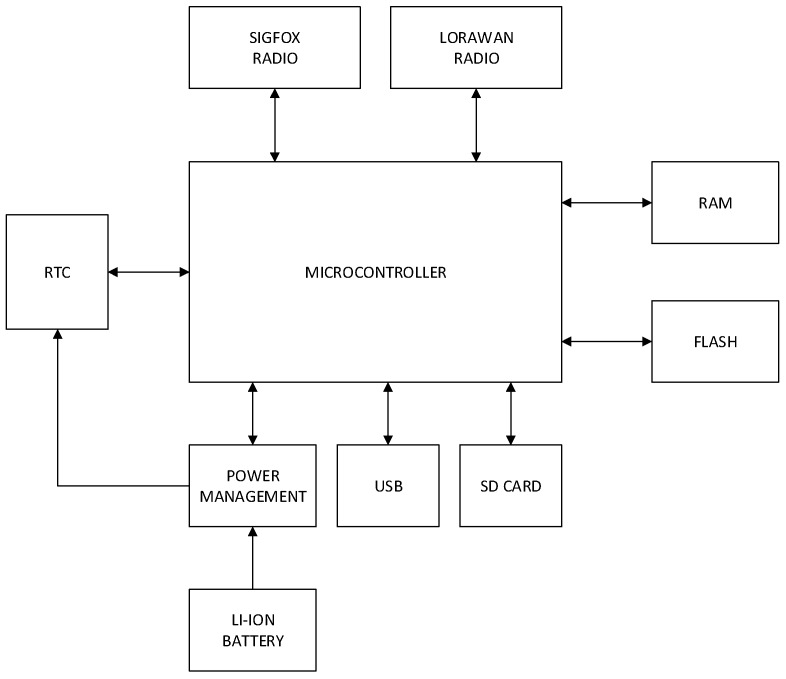
Gateway block diagram.

All the elements that are part of this design are shown in the block diagram of Figure 17. The set is protected by a box made of ABS plastic with IP54 protection degree and antennas and power supply connectors located on the sides.

### 4.4. LoRaWAN Base Station

**Figure 18:**
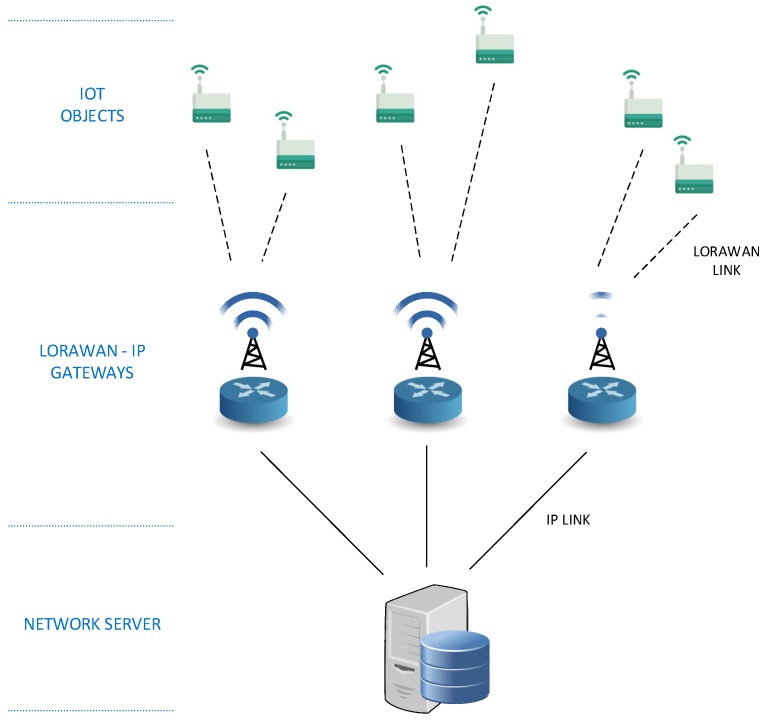
LoRaWAN network architecture.

The purpose of the device described here is to act as gateway between LoRaWAN in 868 MHz ISM radio band and a wired or wireless 2.4 GHz IP network. LoRaWAN is used by the final IoT devices and in the IP network are located the LoRaWAN network server responsible for managing the communication with devices and the application server responsible for managing the information received from devices. Both the typical architecture of a LoRaWAN network and the situation in it of the base station can be seen in Figure 18.

**Figure 19:**
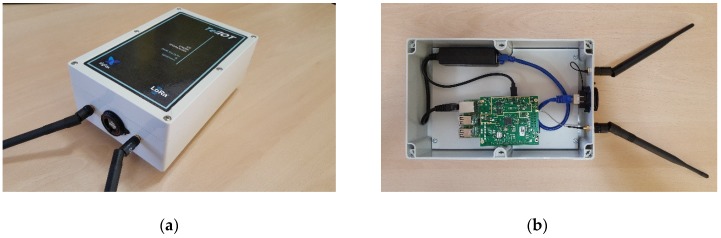
LoRaWAN base station: (**a**) External view; (**b**) Internal view.

The cost is lower than other commercial solutions with the same functionality. As an estimation, a commercial product such as MultiConnect Conduit IP67 from Multitech has a market price around € 1400 (July 2018). The proposed solution costs 396€. Although it does not have all the functionalities of the commercial product, it presents enough to cover the needs of work in the case studies analysed, which makes it a competitive product compared to the previous one. In addition, it has the advantage of using open-source software that can be improved and customized without relying on third parties. Unlike other low-cost solutions, it does not perform a LoRa point-to-point link with a single device simultaneously, but is fully compatible with the LoRaWAN specification and thus with the possibility of receiving several frequencies simultaneously, linking several devices at the same time and taking advantage of extended capabilities such as the adaptive bit rate to achieve optimal link quality in each case. Power is supplied using POE (Power Over Ethernet) to avoid additional wiring and the whole has been assembled in a IP67 box with external antennas and pole mounting bracket. The main controller is based on Raspberry Pi in order to reduce costs and use open-hardware. For the LoRaWAN connectivity, the IMST ic880A board [39] is used, which includes a LoRaWAN SX1301 concentrator and a double SX1257 radio capable of handling up to 8 simultaneous 125 kHz channels. Due the ic880A board does not have the proper format to be directly connected to Raspberry Pi, it has been necessary to insert a connection adapter card, allowing communication between them via the SPI bus. About software, the packages lora_gateway and packet_forwarder [40] have been used on Raspbian OS and they have been configured with the IP address of the available LoRaWAN network server. This hardware and software configuration facilitates scalability according to the needs of each application case, assuming an advantage over closed commercial solutions. The prototype shown in Figure 19 could evolve to a single card format containing both the LoRaWAN radio and concentrator chips, and the processor and communications chipset used in the Raspberry Pi board. Furthermore, in Figure 19b you can see an additional external antenna with the idea of expanding the base station with a Sigfox radio module that would be connected to the UART (Universal Asynchronous Receiver Transmitter) port of Raspberry Pi.

## 5. Case Study and Testing Environment

**Figure 20:**
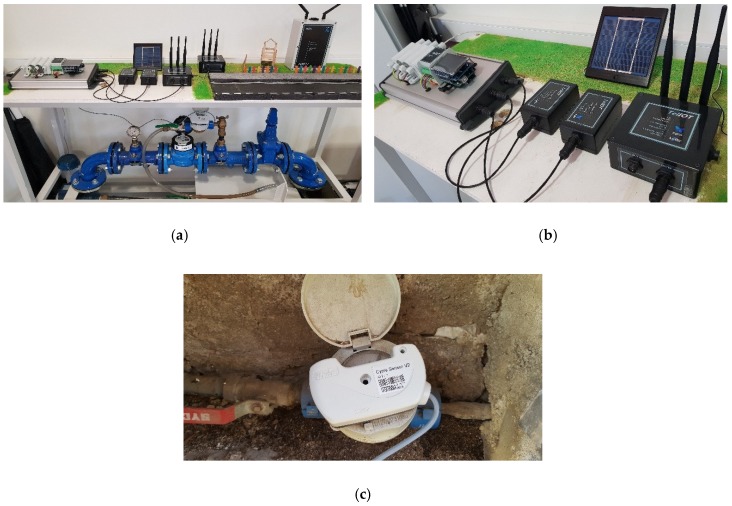
Testing system: (**a**) Mock-up of the system model consisting of: closed water circuit, electric pump, residential water-meter and electronic pulse generator; (**b**) Set of hardware devices developed for remote monitoring, LoRa-Sigfox gateway and test console containing: water-meter emulator, switching device between real water meter or emulator, electronic pulse generator and commercial IoT tele-operated products. (**c**) Electronic pulse generator device attached to a company water-meter placed at the front wall of a residential house.

The validation of the developed system has been carried out in two different phases. A first phase has been performed at the laboratory level using a mock-up of the system model (Figure 20a), which consists of a closed water circuit, an electric pump, a residential water-meter similar to that used by the distribution company and an electronic-pulse generator device as shown in Figure 20c, which is attached to the water-meter, whose output is connected to the input connector of the acquisition device described in Section 4.2.

Figure 20b shows the final versions of each of the different devices developed and described in the previous sections that have been tested during this laboratory phase.

The main goal of this testing phase was to verify that all the hardware worked properly, that all the electrical pulses coming from the water-meter were acquired rightly and that all the necessary information was transmitted without losses by the network in an appropriate way, in order to assure that all developed devices presented an appropriate behaviour, at both hardware and software level, and they could be considered fully operational final prototypes.

Once the testing phase has been passed, a second phase was implemented, consisting of the development of a case study under real operating conditions. For this purpose, it was made an application to a commercial water distribution company of a set of data remotely acquired from several of its water-meters. The remote acquisition system used by the company is based on a 169 MHz radio link communication within the ISM Band. The reception antenna is located on the roof of a building that the company has in the city of Cartagena. The acquisition system is configured in such way it captures and transmits a measurement every hour, so the full monitoring process of a day would involve the acquisition and transmission of 24 samples.

**Figure 21:**
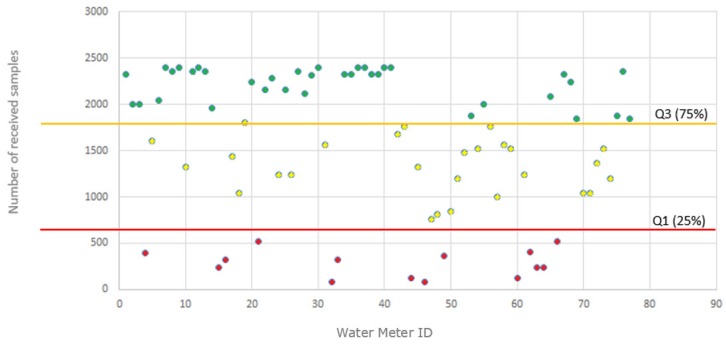
Number of samples received via radio (169 MHz ISM band) from 78 counters randomly selected from different areas of the city.

The company supplied the measurements acquired during a 100-day period, coming from a set of 78 water-meters located in different areas of the city chosen randomly. Figure 21 summarizes the data gathered by these water-meters. Being samples acquired over 100 days, a total amount of 2400 samples should theoretically have been received from each water-meter (100 days × 24 samples/day). However, as shown in Figure 21, this has not happened for all the water-meters, which implies that there are problems associated with the data-transmission process. Using statistical quartiles Q1 (25%) and Q3 (75%), the range of received samples has been divided into three different zones. All those water-meter from which have been received an amount of measurements less than 25% of the expected quantity during the considered monitoring period have been classified as ‘*alarming*’ (marked in red in the figure). Those water-meters from which have been received an amount of measurements between the 25% and 75% of the expected quantity, have been classified as ‘*critical*’ (marked in yellow in the figure). Finally, those water-meters from which an amount of received measures is greater than 75% of the expected amount, have been considered as ‘*acceptable*’ (marked in green).

An ulterior analysis of the location of the water-meters, showed that samples received from those below the quartile 25% come mostly from devices placed in basements of buildings, which suggests that the missing-values are mainly associated with the fact that the radio signal has not enough power to pass through the structure of the building. Samples from water-meters between the quartiles of 25% and 75% present a greater diversity associated with their origin. Some are located in basements, with perhaps better radio coverage and others are located on ground floors or entrances to residential homes in the open, suggesting that they may be located in ‘shadowed’ reception areas due to the fact that they are in an urban environment in which radio coverage can present a certain variability.

It is important to highlight that the company carries out a periodic campaign in which qualified personnel write down ‘in situ’ the consumption data indicated by the water-meter. Therefore, so for billing purposes this loss of samples does not influence the generation of the invoice for the customer and has no economic impact in what is charged as a service by the distribution company. However, these data losses do influence the strategic and operational areas of the company, not only because it implies that it is necessary to maintain a person dedicated to a manual inspection, but it also makes impossible to estimate the consumption habits of some users. This could be possible with no transmission losses due to the fact that although the current water-meters are configured to send a sample every hour, the sampling time could be reduced up to 15 min.

A case study has been designed in which 5 buildings have been selected from the set with water-meters with a sample reception rate less than 25%, 3 buildings with water-meters located in critical areas (25%–75%) and 3 buildings located in the acceptable zones. In these buildings, in parallel with the current company water-meter based on the 169 MHz ISM Band, a prototype of each class (Sigfox, LoRaWAN and LoRa-Sigfox Gateway) has been placed. The LoRa-Sigfox Gateway device has been placed at the light yard of the buildings.

A total amount of 33 acquisition prototypes have been deployed: 11 Sigfox, 11 LoRaWAN and 11 LoRa-Sigfox Gateway. In parallel, a 169 MHz ISM-based monitoring device has been left in order to compare transmission data rates coming from those currently used by the company with the transmission rates associated with each developed prototype. The results after 10 consecutive monitoring days are described below.

**Figure 22:**
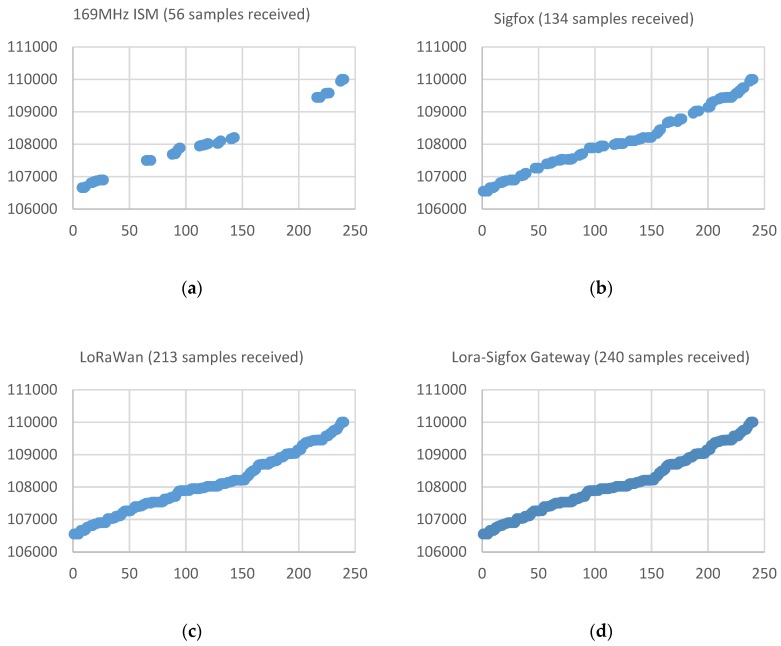
Number of samples received in the case of a building in which the reception rate is below the Q1 quartile (25%). (**a**) In the 169 MHz ISM band, 56 samples were received (23.3%). (**b**) Reception rate with the Sigfox prototype of 55.8% (**c**) Reception rate with the LoRaWan device of 88.8% (**d**) Reception rate with the LoRa-Sigfox Gateway device of 100%.

Figure 22 shows a graph with the samples received during a period of 10 days (which theoretically it should be equivalent to an amount of 240 received samples) by each of the different acquisition devices placed in one of the buildings associated with one of the so-called ‘alarming’ water-meters (Q1 < 25%). This water meter is located in the basement of a residential building. The X axis indicates the number of the sample associated with the time at which it was acquired (values between 1 and 240 for the 10-day evaluation period with a sampling time of one hour) and the Y axis indicates the value (litres) measured by the water meter and transmitted by the acquisition device.

This figure shows that according to the ‘critical’ classification previously indicated, the samples received with the device operating at the 169 MHz ISM Band, instead of the expected 240, is only 56, which means a reception rate of 25%. With the Sigfox prototype, an improvement in the reception rate is achieved, reaching the 134 samples received (55.8%). The LoRaWAN prototype receives 213 samples (88.8%) during the period. The LoRaWAN base station is located closer to the water-meter placement than the Sigfox station, whose location is determined by the official network operator for the city of Cartagena. The results obtained for this deployment seems to indicate that the problem associated with reception is due to the fact that the signal presents a strong attenuation and it is difficult to reach the physical location of the water-meter. Figure 22d shows that the solution with a LoRa-Sigfox Gateway prototype allows to obtain a signal power that allows penetrating and reaching the basement of the building, achieving a reception rate of 100%.

Table 1 summarizes the results of the study case for each of the different buildings with acquiring devices attached to water-meters placed on locations where reception rate in the 169 MHz ISM band was less than 25%. According to the received data, it is confirmed that the 169 MHz ISM band continues to present data losses in the reception. Solutions based on Sigfox and LoRaWAN improve reception. The LoRaWAN prototype, with a base station placed closer to the water-meter than Sigfox, presents a higher reception rate, which seems to reinforce the idea that the losses are due to the fact that the radio signal is not able to reach the location of the water-meter. Finally, in the LoRa-Sigfox Gateway solution it is possible to achieve a 100% reception. Consequently, for water-meters located in this range where reception is less than 25%, the LoRa-Sigfox Gateway solution seems to be the most suitable if you want to achieve a reception rate of 100%.

**Figure 23:**
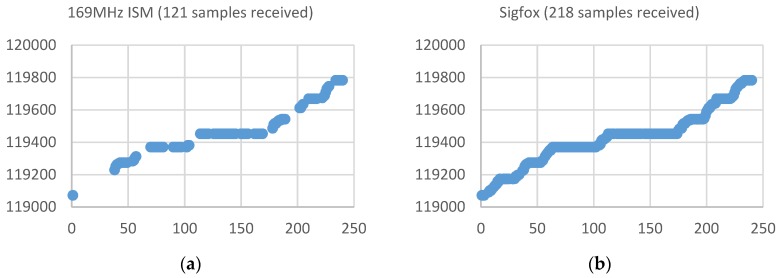

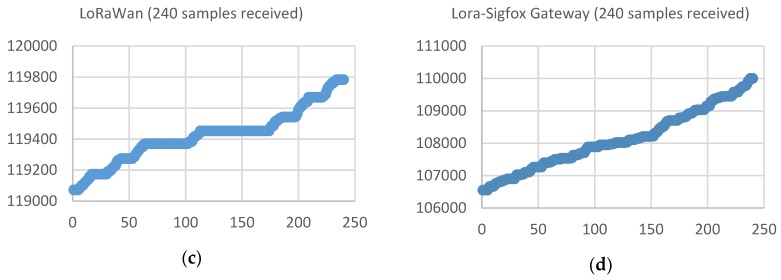
Number of samples received in the case of a building in which the reception rate is ranged within 25%-75%. (**a**) In the 169 MHz ISM band, 121 samples were received (50.4%). (**b**) Reception rate with the Sigfox prototype of 90.8% (**c**) Reception rate with the LoRaWan device of 100% (**d**) Reception rate with the LoRa-Sigfox Gateway device of 100%.

Figure 23 shows a graph with the samples received during the same period of 10 days (240 samples) by each of the different acquisition devices placed in one of the buildings associated with one of the so-called ‘critical’ water-meters (reception rate within 25%–75%). This water meter is located in the basement of a residential building. The X axis indicates the number of the sample associated with the time at which it was acquired (values between 1 and 240 for the 10-day evaluation period with a sampling time of one hour) and the Y axis indicates the value (litres) measured by the water meter and transmitted by the acquisition device.

This figure shows that the samples received with the device operating at the 169 MHz ISM Band, instead of the expected 240, is now 121, which means a reception rate of 50.4%. With the Sigfox prototype, an improvement in the reception rate is achieved, reaching the 218 samples received (90.8%). In this case, both the prototype based on LoRaWAN and the prototype LoRa-Sigfox Gateway presents a signal strength that allows penetrating and reaching the basement of the building, achieving a reception rate of 100%. In this case, it seems that since the LoRaWAN base station is closer to the water-meter than the Sigfox, the LoRaWAN coverage ensures an adequate reception rate.

Table 2 summarizes the results of the study case for each of the different buildings with acquiring devices attached to water-meters placed on locations where reception rate in the 169 MHz ISM band was ranged within 25%–75%. It can be noted that Sigfox improves the reception rate, reaching in some cases a reception rate of 100%. However, it is the solutions based on LoRaWAN and LoRa-Sigfox Gateway that ones that achieve a reception rate of 100% in every analyzed building. In the case of LoRaWAN, possibly the main reason is due to the fact that its base station is placed closer to the water-meter than the Sigfox, which current location is determined the operator of the area. 

**Figure 24:**
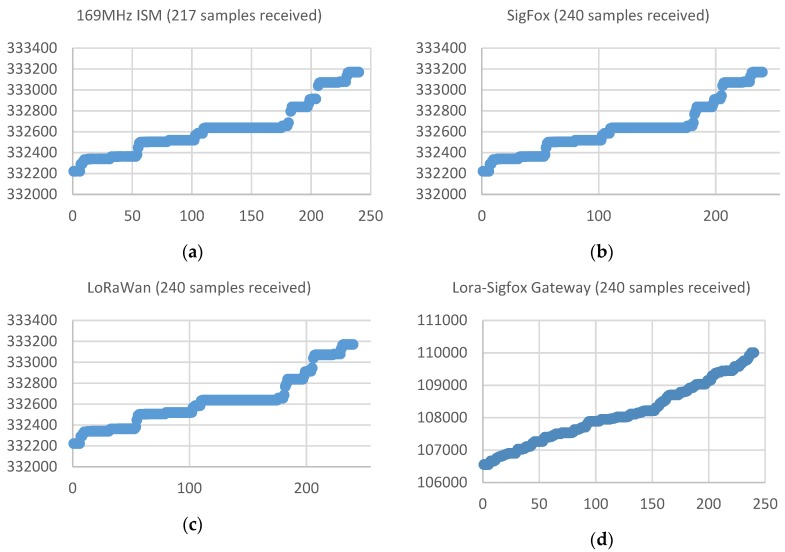
Number of samples received in the case of a building in which a reception rate higher than 75%. (**a**) In the 169 MHz ISM band, 217 samples were received (90.4%). (**b**) Reception rate with the Sigfox prototype of 100%. (**c**) Reception rate with the LoRaWan device of 100%. (**d**) Reception rate with the LoRa-Sigfox Gateway device of 100%.

Figure 24 shows a graph with the samples received during the same period of 10 days (240 samples) by each of the different acquisition devices placed in one of the buildings associated with one of the so-called ‘acceptable’ water-meters (reception rate higher than 75%). This water-meter is located in the entrance box to a residential house (chalet), similar to the one showed in Figure 20c. The X axis indicates the number of the sample associated with the time at which it was acquired (values between 1 and 240 for the 10-day evaluation period with a sampling time of one hour) and the Y axis indicates the value (litres) measured by the water meter and transmitted by the acquisition device.

This figure shows that the samples received with the device operating at the 169 MHz ISM Band, instead of the expected 240, is now 217, which means a reception rate of 90.4%. In this case, the three tested prototypes achieve a 100% reception-rate.

Table 3 summarizes the results of the study case for each of the different buildings with acquiring devices attached to water-meters placed on locations where reception rate in the 169 MHz ISM band is higher than 75%. As said before, the three tested prototypes achieve a 100% reception-rate, which clearly confirms that if there are no shadow zones in coverage, the IoT technologies improve the performance of classic solutions such as the 169 MHz ISM Band used by the water distributor.

To check system performance and ensure load capacity, the whole set has been subjected to stress testing above the common operating conditions. Instead of sending data once an hour, all the devices have been configured to send data every 10 min, the minimum allowed interval due to duty cycle regulation in the 868 ISM band. In a period of 72 h, it supposes 432 frames per device and a total of 14256 frames, equivalent to 99 days sending data once an hour. It allows check hardware devices reliability and system behaviour in a longer operational period. Reception rates can be seen in Table 4, very close to those obtained in the 10 days period study case.

Increasing the number of devices and so on the network load, is not a problem for Sigfox and LoRaWAN technologies due are able to manage 10^6^ and 10^4^ devices per base station respectively. About our own LoRa-Sigfox gateway, the number of end devices using a single unit in a real scenario is estimated to be no more than 10, so 11 of them where used in the testing environment with success of 0% data loss. Nevertheless, a stress test was conducted in laboratory simulating 150 different devices sending data through the same gateway. A special firmware loaded in three meter data acquisition devices allowed to send frames at random intervals with different device identifiers for 24 h. The result was a 99.1% success rate probably due to frame collisions or RF noise. Because the water consumption data sent is accumulative, a 0.9% frame loss is acceptable in this application. 

**Figure 25:**
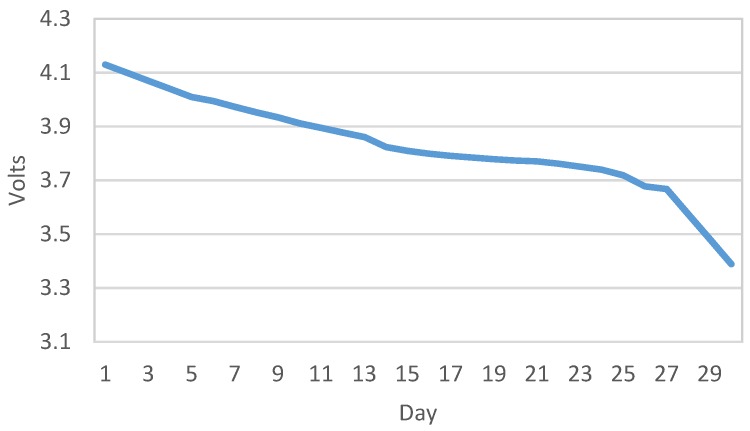
Battery voltage evolution.

Furthermore, in order to check reliability and autonomy of the meter acquisition device, it has been configured to send data continuously every 10 min. Battery voltage evolution and consequently state of charge can be seen in Figure 25. Sending 144 frames every day, the daily voltage drop is 0.73 V and it reach the minimum device operational voltage in day 29. It allows us to estimate autonomy of 9 years sending 1 frame every day.

## 6. Conclusions

This article has been focused on the design and implementation of a mixed IoT LPWAN network architecture with application for remote monitoring in urban environments, where shadowed areas associated radio coverage can arise. LPWAN is described as a promising technology that allows deployment of wide area and low power networks specific to IoT. In particular, the approach has been to concentrate on Sigfox and Lora technologies because they allow the deployment of a large number of connected objects at a low infrastructure costs. They offer long-range connectivity to cheap, battery-operated devices, which require the transmission of small amounts of data at regular intervals of time over a long lifespan.

A set of mixed IoT LPWAN end-devices has been developed. Its design has been oriented to acquire water-meter consumption measurements. A mixed network architecture dedicated to ensure all acquired samples are transmitted and successfully received at the remote information system has been proposed too. A specific LoRa-Sigfox Gateway device has been designed and included in the proposed network. This device is capable of take advantage of the LoRa basis in order to have a strong signal capable of connect to an end-device located at places with bad coverage, and then transform the data in order to send it and connect in a transparent way with a Sigfox network, already deployed by an existing network operator that finally transmits data towards the final reception systems.

A study case of a system architecture connected to a set of 11 different buildings located at places with different coverage levels has been performed. From the results obtained in this deployment it is possible to conclude that:

- In locations where the coverage in the 169 MHz ISM band has a reception rate lower than 25%, it seems that a solution like the one described in the LoRa-Sigfox Gateway prototype allows an optimal reception rate (100%) to be ensured. The Sigfox or LoRaWAN technologies improve reception, but do not achieve 100% reception rate, probably due to losses in signal strength that avoids a good penetration towards the location of the water-meters.
- In locations where the reception rate in the 169 MHz ISM band is ranged within 25% and 75%, the LoRa-Sigfox and LoRaWAN prototypes offer the best performance. This seems to indicate that communication with the Sigfox station, whose location is predetermined by the network operator in the area, sometimes does not have enough power to penetrate to the location of the water-meter. Therefore, it would be necessary to evaluate one of the following alternative solutions: (1) placing a private (owned by the water distribution company) LoRaWAN base station on a building’s roof within the radius of action of the water-meters and then place a LoRaWAN acquisition device attached to in each water meter, or (2) a solution based on two devices, a LoRa-Sigfox Gateway acquisition device plus the Gateway itself placed at the light yard of the house, which transmits the signal to an area where there is suitable Sigfox connectivity and can communicate with the local network operator station.
- In areas where the reception rate in the 169 MHz ISM band is greater than 75%, the three prototypes allow reaching a 100% reception rate. In this case, the most suitable solution seems to be the solution based on the Sigfox prototype, since it would not be necessary to deploy any additional base station, as is required by the solution based on the LoRaWAN prototype, and only one device is necessary, instead of the two devices (end device + gateway) associated with the solution based on the LoRa-Sigfox Gateway prototype.
- An additional improvement over the solution given by the current device working in the 169 MHz ISM band is that the three prototypes developed can be configured to have a sampling rate up to 10 min, compared to the resolution limit of 15 min offered now by the ISM solution, thus improving not only the rate of reception, but also by expanding the resolution of the sampling.
- As the proposed solutions ensures an acquisition rate of 100%, it is possible to avoid the need for having qualified personnel moving monthly to manually hand write measurements from each water-meter, being their action only necessary in specific cases or in periodic revision situations in order to confirm the correct operation of the acquisition devices.
- Finally, it would allow the distribution company to have a greater number of measures on which ‘Big Data’ [41] techniques could be applied in order to estimate the consumption habits of the users and to foresee future consumption peaks, improving the strategies of maintenance and expansion of the distribution network of the company.

## Figures and Tables

**Table 1:** Reception rates over a period of 10 days (240 samples) with each of the different systems tested in a water-meter with a percentage of received samples ranged lower than 25% over expected in the 169 MHz ISM band.

Building	169 MHz ISM	Sigfox	LoRaWAN	LoRa-Sigfox Gateway
B01	56 (23%)	134 (55.8%)	213 (88.8%)	240 (100%)
B02	36 (15%)	101 (42.1%)	166 (69.2%)	240 (100%)
B03	32 (13.3%)	92 (38.3%)	173 (72.1%)	240 (100%)
B04	41 (17.08%)	110 (45.8%)	188 (78%)	240 (100%)
B05	24 (10)	89 (37.1%)	146 (60.1%)	240 (100%)

**Table 2:** Reception rates over a period of 10 days (240 samples) with each of the different systems tested in a water-meter with a percentage of received samples ranged within 25%–75% over expected in the 169 MHz ISM band.

Building	169 MHz ISM	Sigfox	LoRaWAN	LoRa-Sigfox Gateway
B06	121 (50.4%)	218 (90.8%)	240 (100%)	240 (100%)
B07	148 (61.7%)	229 (95.4%)	240 (100%)	240 (100%)
B08	170 (70.8%)	240 (100%)	240 (100%)	240 (100%)

**Table 3:** Reception rates over a period of 10 days (240 samples) with each of the different systems tested in a water-meter with a percentage of received samples ranged higher than 75% over expected in the 169 MHz ISM band.

Building	169 MHz ISM	Sigfox	LoRaWAN	LoRa-Sigfox Gateway
B09	217 (90.4%)	240 (100%)	240 (100%)	240 (100%)
B10	187 (77.9%)	240 (100%)	240 (100%)	240 (100%)
B11	198 (82.5%)	240 (100%)	240 (100%)	240 (100%)

**Table 4:** Reception rates over a period of 72 h sending data every 10 min (14256 frames).

Device Type	Devices	Received Frames	Expected Frames	Success Rate
SIGFOX	11	2623	4752	55.2%
LoRaWAN	11	4143	4752	87.2%
LoRa-Sigfox	11	4728	4752	99.5%

## References

[1] Rahman A.F.A., Daud M., Mohamad M.Z. Securing sensor to cloud ecosystem using internet of things (IoT) security framework. Proceedings of the International Conference on Internet of things and Cloud Computing, ACM.

[2] Lee I., Lee K. (2015). The Internet of Things (IoT): Applications, investments, and challenges for enterprises. Bus. Horiz..

[3] International Telecommunication Union (2005). ITU Internet Reports 2005: The internet of Things.

[4] Ganguli S., Friedman T. IoT Technology Disruptions: A Gartner Trend Insight Report. https://www.gartner.com/doc/3738060/iot-technology-disruptions-gartner-trend.

[5] Yang W., Wang M., Zhang J., Zou J., Hua M., Xia T., You X. (2017). Narrowband Wireless Access for Low-Power Massive Internet of Things: A Bandwidth Perspective. IEEE Wirel. Commun..

[6] Davoli L., Belli L., Cilfone A., Ferrari G. (2018). From Micro to Macro IoT: Challenges and Solutions in the Integration of IEEE 802.15.4/802.11 and Sub-GHz Technologies. IEEE Internet Things J..

[7] Sanchez-Iborra R., Cano M.-D. (2016). State of the Art in LP-WAN Solutions for Industrial IoT Services. Sensors.

[8] Lloret J., Tomas A., Canovas A., Parra L. (2016). An integrated IoT architecture for smart metering. IEEE Commun. Mag..

[9] Fremantle P. (2015). A Reference Architecture for the Internet of Things.

[10] Stewart R.A., Willis R., Giurco D., Panuwatwanich K., Capati G. (2010). Web-based knowledge management system: Linking smart metering to the future of urban water planning. Aust. Planner.

[11] Voulvoulis N., Arpon K.D., Giakoumis T. (2017). The EU Water Framework Directive: From great expectations to problems with implementation. Sci. Total Environ..

[12] Montginoul M., Vestier A. (2018). Smart metering: A water-saving solution? Consider communication strategies and user perceptions first. Evidence from a French case study. Environ. Modell. Softw..

[13] Raza U., Kulkarni P., Sooriyabandara M. (2017). Low Power Wide Area Networks: An Overview. IEEE Commun. Surv. Tutorials.

[14] Khutsoane O., Isong B., Abu-Mahfouz A.M. IoT devices and applications based on LoRa/LoRaWAN. Proceedings of the IECON 2017—43rd Annual Conference of the IEEE Industrial Electronics Society.

[15] Mekki K., Bajic E., Chaxel F., Meyer F. (2018). A comparative study of LPWAN technologies for large-scale IoT deployment. ICT Express.

[16] European Research Council ERC Recommendation 70-03 Relating to the use of Short Range Devices. https://www.ecodocdb.dk/download/25c41779-cd6e/Rec7003.pdf.

[17] SIGFOX Company. https://www.sigfox.com.

[18] Sigfox M2M and IoT Redefined through Cost Effective and Energy Ptimized Connectivity. https://lafibre.info/images/3g/201302_sigfox_whitepaper.pdf.

[19] Centenaro M., Vangelista L., Zanella A., Zorzi M. (2016). Long-range communications in unlicensed bands: The rising stars in the IoT and smart city scenarios. IEEE Wirel. Commun..

[20] Sigfox Sigfox Certification Handbook. https://build.sigfox.com/steps/certification/#what-is-a-sigfox-certification.

[21] Do M.T., Goursaud C., Gorce J.M. Interference Modelling and Analysis of Random FDMA Scheme in Ultra Narrowband Networks. Proceedings of the Tenth Advanced International Conference on Telecommunications, AICT 2014.

[22] Lauridsen M., Vejlgaard B., Kovacs I.Z., Nguyen H., Mogensen P. Interference Measurements in the European 868 MHz ISM Band with Focus on LoRa and SigFox. Proceedings of the 2017 IEEE Wireless Communications and Networking Conference (WCNC).

[23] Sigfox Radio Technology Keypoints. https://www.sigfox.com/en/sigfox-iot-radio-technology.

[24] Sigfox Sigfox Technology Overview. https://www.sigfox.com/en/sigfox-iot-technology-overview.

[25] Vejlgaard B., Lauridsen M., Nguyen H., Kovacs I.Z., Mogensen P., Sorensen M. Coverage and Capacity Analysis of Sigfox, LoRa, GPRS, and NB-IoT. Proceedings of the 2017 IEEE 85th Vehicular Technology Conference (VTC Spring).

[26] Technical Overview of Sigfox Technology: Network Architecture, Interfaces, Protocol Stack. https://www.survivingwithandroid.com/2018/07/sigfox-protocol-network-architecture-iot-protocol-stack.html.

[27] Semtech Corporation LoRa Overview. https://www.semtech.com/lora.

[28] Sanchez-Iborra R., Sanchez-Gomez J., Ballesta-Viñas J., Cano M.-D., Skarmeta A. (2018). Performance Evaluation of LoRa Considering Scenario Conditions. Sensors.

[29] Semtech LoRa™ Modulation Basics AN1200-22. https://www.semtech.com/uploads/documents/an1200.22.pdf.

[30] Feltrin L., Buratti C., Vinciarelli E., De Bonis R., Verdone R. (2018). LoRaWAN: Evaluation of Link- and System-Level Performance. IEEE Internet Things J..

[31] LoRa Alliance LoRaWAN Specification v1.1. https://lora-alliance.org/resource-hub/lorawantm-specification-v11.

[32] Adelantado F., Vilajosana X., Tuset-Peiro P., Martinez B., Melia-Segui J., Watteyne T. (2017). Understanding the Limits of LoRaWAN. IEEE Commun. Mag..

[33] Robert Miller MWR Labs Whitepaper, LoRa Security Building a Secure LoRa Solution. https://labs.mwrinfosecurity.com/assets/BlogFiles/mwri-LoRa-security-guide-1.2-2016-03-22.pdf.

[34] Margelis G., Piechocki R., Kaleshi D., Thomas P. Low Throughput Networks for the IoT: Lessons learned from industrial implementations. Proceedings of the IEEE World Forum on Internet of Things, WF-IoT 2015.

[35] Pycom LoPy4 Development Board Datasheet. https://docs.pycom.io/datasheets/development/lopy4.

[36] Pycom Company. https://pycom.io.

[37] MicroPython Community MycroPython Website. https://micropython.org.

[38] Libelium Waspmote Technical Overview. http://www.libelium.com/products/waspmote/hardware.

[39] IMST GmbH iC880A LoRaWAN Concentrator 868 MHz Datasheet. https://wireless-solutions.de/downloads/Radio-Modules/iC880A/iC880A_Datasheet_V1_0.pdf.

[40] GitHub Driver/HAL to Build a Gateway using a Concentrator Board Based on Semtech SX1301 Multi-Channel Modem and SX1257/SX1255 RF Transceivers. https://github.com/Lora-net/lora_gateway.

[41] Djedouboum A.C., Abba Ari A.A., Gueroui A.M., Mohamadou A., Aliouat Z. (2018). Big Data Collection in Large-Scale Wireless Sensor Networks. Sensors.

